# Are we preparing healthy & responsible adolescents? Exploratory qualitative study to understand the health and social issues of adolescent living in Karachi, Pakistan

**DOI:** 10.1371/journal.pgph.0003736

**Published:** 2024-10-15

**Authors:** Narjis Rizvi, Sarah Saleem, Jawaria Mukhtar Ahmed, Sayyeda Ezra Reza, Rawshan Jabeen, Saleem Jessani

**Affiliations:** Community Health Science CHS, Aga Khan University, Karachi, Pakistan; PLOS: Public Library of Science, UNITED STATES OF AMERICA

## Abstract

This study aimed to understand the lives of adolescents living in squatter settlements of Karachi, Pakistan regarding their assigned roles and responsibilities, health and social issues, their decision-making process, and mechanism and channels of information. An exploratory descriptive qualitative study design was employed. Purposive sampling techniques were used to carry out Focus Group Discussions (FGDs) with adolescents (n = 10, Participants = 190), adolescents’ parents (n = 10, Participants = 180), and In-Depth Interviews of with key adolescent stakeholders (n = 20). Adolescent stakeholder mapping was conducted for enrolling participants. The data were analysed thematically using deductive and inductive approaches. Based on gender norms parents assign specific roles and responsibilities to adolescent girls and boys. Due to societal norms, communication gap exists between adolescents and their parents. The most popular information channels are social media platforms and friends. Adolescents reported being subjected to a variety of physical, sexual, mental, social, and environmental pressures. Lack of guidance from parents, inadequate knowledge and skills to deal with physical, sexual, mental, social and environmental hazards, and misuse of social media lead to risky decisions, injuries, and social instability. This study underlines the urgent need for targeted interventions for addressing gender issues and improving adolescents’ decision-making and life skills. We recommend Behavior Change Communication interventions to dismantle gender stereotypes and support a balanced domestic environment for children’s education and well-being, awareness raising among parents, teachers, and healthcare providers about adolescent risks emphasizing their role in youth guidance, and advocacy for youth-led forums to co-create educational content engaging parents, educators, and health professionals focusing on life skills. These strategies would turn demographic transitions into productive dividends.

## Background

The World Health Organization (WHO) defines adolescence as being between the ages of 10 and 19 years [1] while the Lancet Commission reports an inclusive age range of 10–24 years [2]. Regardless of age, adolescence is the transition period between childhood and adulthood [2]. During this phase, adolescents perform and experience many tasks for the first time including the acquisition of the emotional and cognitive abilities for independence, completion of education, initiation of employment, social engagement, taking responsibilities, formation of lifelong relationships, choosing a spouse, marriage, sexual debut, beginning of a family, and parenthood. As a result, this transition provides numerous opportunities for physical, social, and emotional growth [3]. However, it also poses several risks to adolescent health and well-being because this is the stage at which gender differences crystallize and shape their roles [3]. Therefore, adolescents must be equipped with the necessary knowledge and skills to promote positive development and effectively manage changing needs, roles, and responsibilities[4].

Adolescents account for 16% of the global population, with 22% living in South-East Asia Region (SEAR). Adolescents have been overlooked in global health and social policy until recently, which is one reason they have seen fewer health gains and economic development than other age groups. Universal Health Coverage (UHC), with the goal of “health for all at all ages,” provides a significant medium for governments to meet adolescents’ health needs and improve their overall development and well-being [1]. The Global Strategy for Women’s, Children’s and Adolescents’ Health presents an excellent opportunity for investment in adolescent health and well-being, with the vision of creating a world in which women, children, and adolescents have the right to health, well-being, development, and the ability to contribute to the development of sustainable societies by 2030 [5]. Nonetheless, achieving the Sustainable Development Goals (SDGs) and targets will require large-scale investments in developing the capabilities of adolescents and young adults [6].

Adolescents account for 22.7% of the population in Pakistan [7, 8]. Surveys and research primarily focus on married adolescents and fertility issues [9]. Only one population-based survey on unmarried adolescents, focusing on menarche/puberty, has been conducted on unmarried adolescents that focused on menarche/puberty [10] The scant literature on unmarried adolescents reveals that either no knowledge is provided to assist them in managing their new roles and responsibilities [11]or that the knowledge provided is insufficient, inappropriate, and delayed [12]. Research also shows that unmarried adolescents, despite their willingness, are forbidden to share and seek advice about their reproductive, mental, and social problems [13] as these are societal taboos [14, 15]. Furthermore, societal norms forbid family elders from discussing sexual health, particularly with unmarried adolescents [16]. Adolescents face barriers for accessing health and social care when they need it due to restrictive legislative frameworks, out-of-pocket service costs, stigma, and community attitudes [6] In the absence of adequate knowledge and timely guidance, adolescents make risky decisions that often have negative consequences for their personality, social interactions, and health, [17]. Furthermore, they have been dealing with a variety of health, sexual, reproductive, and social issues [13], which cause confusion and stress [18]. This emphasizes the importance of developing Sexual and Reproductive Health and Rights (SRHR) policies that are relevant to adolescents’ health and social issues and, needs, and transformation of these policies into effective programs [19]. Hence, it is critical to understand the health and social needs of Pakistani adolescents.

In Karachi, Pakistan, squatter settlements lack basic amenities such as health, education, sports, and other social services and infrastructure which are essential for adolescents’ normal growth and upbringing. This study aimed to understand adolescents’ health and social issues, roles, responsibilities, their decision-making process, and mechanism and channels of information in Karachi, Pakistan.

## Methods

### Study design & purpose

A qualitative descriptive study design was employed to understand the lives of adolescents through exploring adolescents, parents and stakeholders’ perspectives by using purposive sampling technique.

### Study setting and participants

- This study was conducted as part of the Sukh Initiative, a collaboration between the Aman, the Bill & Melinda Gates, and the David & Lucile Packard Foundations. This project was implemented by a consortium of six national and international organizations including the Community Health Sciences (CHS) Department of Aga Khan University (AKU). The primary objective of ‘Sukh’ was to increase contraceptive use by 15% among Karachi, Sindh, Pakistan’s 100 million underserved peri-urban population. Additionally, the project also explored the lives of adolescents.
- The study was conducted in ten squatter settlements in four towns of Karachi where the ‘Sukh’ field-based health and social centers were established (Refer to Table 1).

**Table 1:** Town and station-wise list of selected areas.

Town	Area	Station
Bin Qasim Town	Shah Latif Town Sector 19/20	1
	Dabla Para Rehri Goth	2
	Umer Marvi Goth	3
Korangi	Bhittai Colony	4
	Ittehad Colony	5
	Area I Korangi No. 5	6
	Sector 33	7
Landhi	Future Colony	8
	Mansehra Colony	9
Malir	Ghazi Town	10

- Participants included, a diverse set of key stakeholders—adolescents aged 16 to 18, adolescent’s parents, government sports and youth affairs officers, political leaders, social activists, Non-Governmental Organization (NGO) administrators, educational leaders from public and private sectors, healthcare professionals, and community health workers. *List of Participants and data collection techniques (Refer to Table 2)*

**Table 2:** List of participants and data collection techniques.

Data Collection Techniques	Participants’ Categories	Numbers of FGDs/IDIs
Focus group discussion	Adolescents	10 (Total number of Participants = 190)
In-depth interviews	Adolescents’ Parents	10 (Total number of Participants = 180)
Key Informant Interviews	Sports & Youth Affairs Officer Local Political Leader Community Leaders Youth NGO Administrators Head of Schools (Public & Private) Head of Madrassas Healthcare Provider Pharmacist Lady Health Worker	20

- A purposive sampling technique, detailed in Table 1, was designed to capture a wide range of perspectives based on gender, geographic location, and social roles, thus providing a comprehensive understanding of the multifaceted adolescent experience in these communities. This diverse participant pool offered varied viewpoints, enriching our study with insights from different socioeconomic, cultural, and professional backgrounds.
- Ethical approval for this study was obtained from the Aga Khan University Ethical Review Committee (AKU-ERC). Written informed consent was provided by all study participants. Informed consent included permission to audio record the interviews and use anonymized quotes.

### Data collection

Using semi-structured guides, Focus Group Discussions (FGDs) with adolescents (16–18 years) and parents, and In-Depth Interviews (IDIs) with key stakeholders were conducted to collect data. The FGD guide was pilot-tested with six participants in one FGD, while the interview guide was pilot-tested with five participants. Both guides included open-ended questions and probes to elucidate perspectives on adolescents’ characteristics, knowledge of pubertal changes, activities they have been involved in, health and social issues they face, their decision-making process, and mechanisms and channels accessed by them to gather information. During discussions, care was taken to avoid stifling dialogue however opportunity was provided for the emergence of new domains.

Data were collected iteratively by researchers of the Sukh project between 1^st^ December 2014 to 31^st^ January 2017 at local community centers for FGDs and offices of participants for IDIs),. To establish rapport, participants were invited in a cordial manner to voluntarily participate in the interview with full assurances of anonymity, privacy, and confidentiality. The parents of the adolescents then gave their informed, written consent for voluntary participation. For adolescents, parental consent was obtained before taking adolescents ascent.

The duration of FGDs and IDIs was 60 minutes and 40 minutes, respectively. All sessions were audio recorded, pseudo-anonymized, and transcribed. The field notes also included participant impressions and emerging topics. Data collection continued until data saturation was reached and no new information was yielded. All data were encrypted, stored, and processed in accordance with applicable data protection regulations. There was no financial reward for participants. The quality of the research was ensured by using COREQ Checklist (Consolidated criteria for Reporting Qualitative research).

### Data analysis

Using inductive approaches, the data was analyzed and coded and sub-themes and themes were developed [20, 21]. Findings were clarified using the one-sheet-of-paper method [22] and codes were assigned to participants: (FGD = Focus Group Discussion participant; KI = Key Informant participant; F = Female; M = Male) and study sites (SS = Squatter Settlement, I-X). The findings of FGDs with adolescents were triangulated with FGDs with adolescents’ parents and KIs with stakeholders. The findings were translated into English while preserving their culturally specific meanings by using QSR-Nvivo 12 software.

### Study rigor

Rigor was maintained throughout the research by ensuring credibility and confirmability [23, 24]. For credibility, member checking was performed which included reconfirming the FGD content with participants. Secondly, peer debriefing was also performed where the researchers shared and discussed the research findings, interpretations, and methodology with peers. To maintain confirmability, the researchers indulged in reflexivity to ensure mitigation of personal views on research process and ensuring adequate audit trail by keeping comprehensive records of the research process, including rough notes and interview transcriptions.

## Results

In total, 10 Focus Group Discussion with adolescents (n = 190 participants) and adolescent’s parents (n = 180) were conducted respectively. In FGDs with adolescents 53% (n = 105,) were females while 44.7% (n = 85,) were males. In FGDs with adolescent’s parents, 55.5% (n = 100,) were mothers while 44.5% were fathers(n = 80,). A total of twenty stakeholders were interviewed.

The *Town and Station-Wise List of Selected Areas is given in Table 1.*

**Fig 1:**
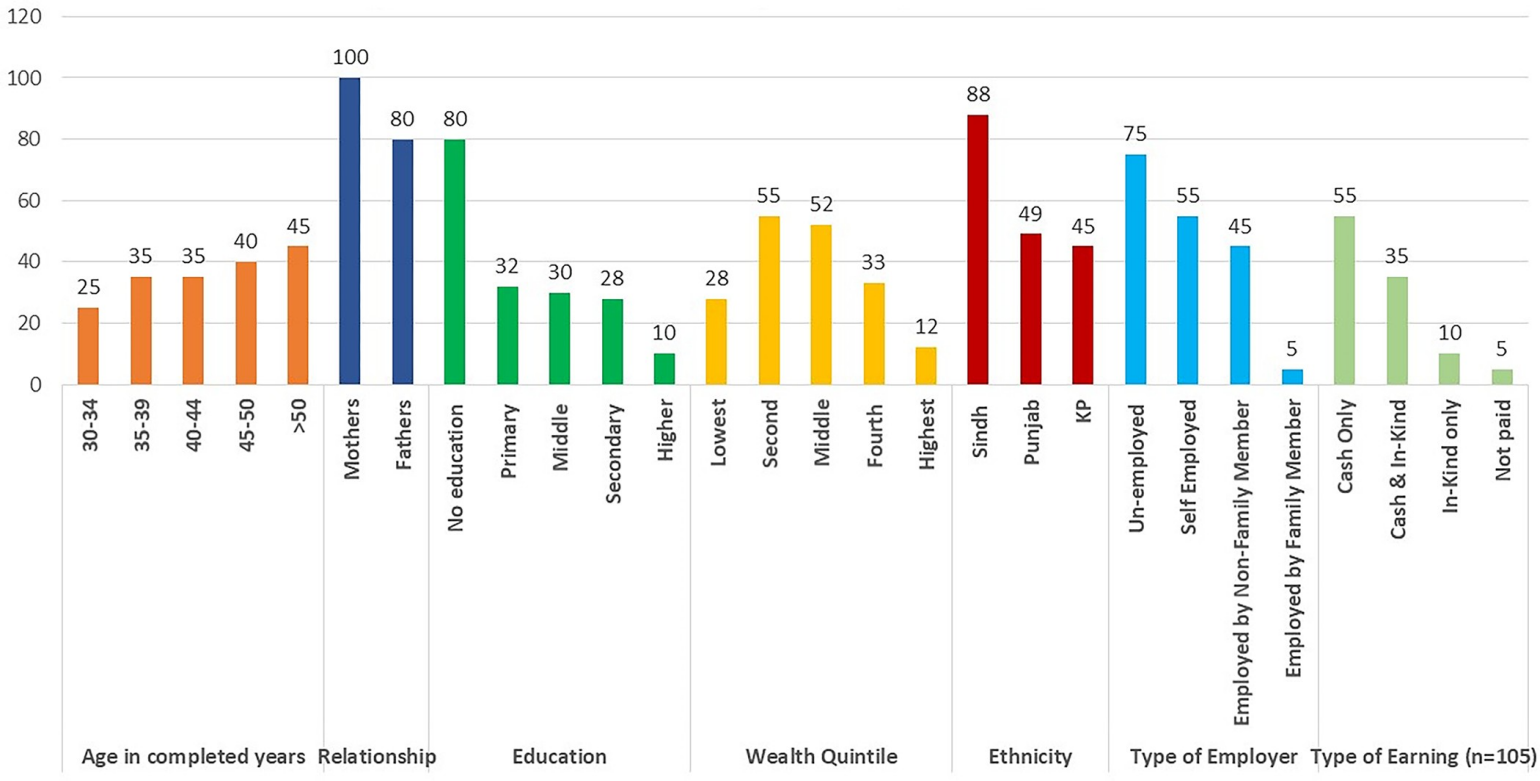
Characteristics of parents (FGD participants n = 180).

**Fig 2:**
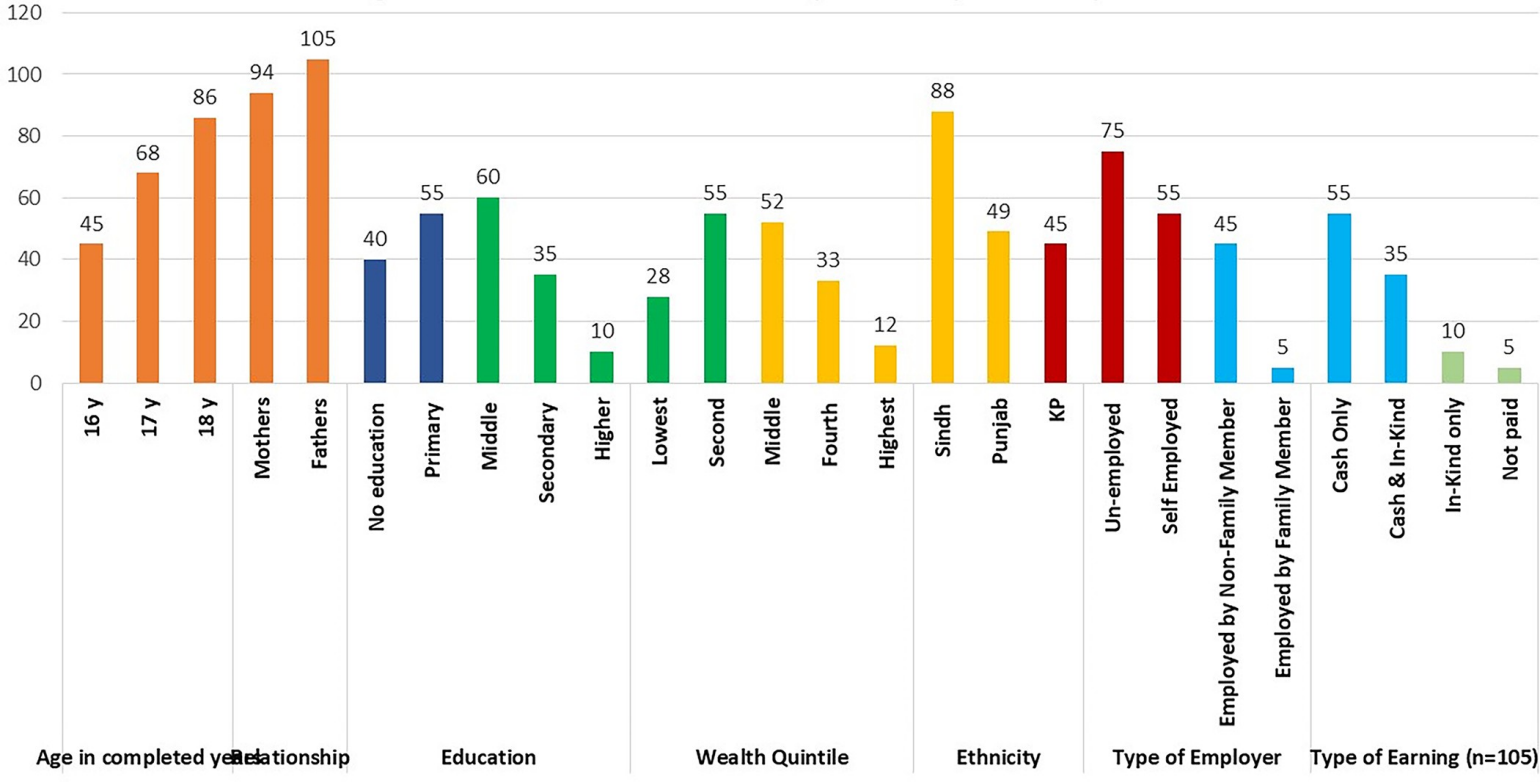
Characteristics demographic of adolescents (FGD participants n = 190).

Data Collection Techniques, Participants Categories and the Number of FGDs/IDIS is given for example the Fig 1 shows Characteristics of Parents (FGD participants n = 180) and Fig 2 shows: Characteristics of adolescents (FGD participants n = 190)

### 1. Adolescents characteristics

Approximately all the adolescents, their parents, and stakeholders believe that the community members are well aware that adolescence begins between the ages of 12 to 14 for girls and 15 to 18 for boys. According to all the respondents, the indicators of the beginning of adolescence are menstruation and breast enlargement in girls and the appearance of mustache, beard, and voice changes in boys. They mentioned:

“Adolescence begins between the ages of 12 and 14 for girls and 15 and 18 for boys and is marked by physical changes.”- *(Adolescents, Parents & Stakeholders)*

### 2. Knowledge about pubertal changes

According to the findings adolescents lack knowledge of pubertal physical changes and are unprepared to manage them. Parents do not inform adolescents about puberty, its associated changes, and the necessary preparations to manage them. Most of the participants reported that because adolescents lack knowledge about pubertal changes, they perceive them as health problems.

"Girls have no prior knowledge of the menstrual cycle; sudden bleeding confuses them, and they believe it is the result of an illness or internal injury."—*(Female Adolescents, Slum I, V & VIII & Lady Health Worker).*

Adolescent males reported that societal norms prohibit adolescents from discussing their pubertal changes and sexual issues and getting guidance.

“Boys cannot discuss their pubertal changes and sexual concerns with anyone as this is unacceptable.”–*(Male Adolescents, SS II, VI & IX & Pharmacist)*

The majority of adolescents of both genders reported that coping with these pubertal changes alone negatively impacts their mental and emotional health.

“We manage our reproductive and sexual issues ourselves, and this give us emotional and mental stress.”–*(Male & Female Adolescents: Slum-I, III, VIII)*

### 3. Adolescent’s role and responsibilities

The participants unanimously reported that based on gender norms parents assign different roles and responsibilities to girls and boys. Girls’ mobility is restricted, they are expected to stay at home and help their mothers with household chores while in a few low-income families’ girls begin paid employment in factories or as domestic servants. Girls believed that social restrictions hinder their education and careers.

“With the onset of menstruation, most of the girls are confined to stay at home and discontinue our education.”–(Female Adolescents: Slum-II, V & IX & School Teacher)

Boys, on the other hand, are allowed to continue their education while few from low-income families engage in productive work by assisting in the family business or working part-time.

Girls and boys engage in different recreational activities. Girls devote some time to self-care, including shopping, dressing up, grooming, visiting relatives, and watching TV. Boys spend the majority of their time playing sports and socializing with their peers outside home. Girls and their mothers reported unequivocally that boy groups verbally and physically harass girls while sitting at hotels or wandering on streets. This places girls at risk for sexual abuse and causes girls and their parents’ stress.

“While sitting in hotels or wandering on the streets, boy’s groups harass girls verbally and physically, which sometimes results in sexual abuse.”- (Mother: Slum II, Female Adolescents: Slum-III, VI, & All Stakeholders)

Even when probed, boys and fathers did not consider that boys’ groups hanging out at small street hotels or wandering the streets could cause harassment for girls.

The ownership and use of mobile phones also differ between girls and boys; more boys own and use cell phones than girls. Mothers agreed that most boys and a few girls misuse their cellphones.

“Boys most of the time are busy playing with their mobile phones; at times, this distracts them from studies and routine work.”- (Mothers: Slum-VI & School Teacher)

### 4. Adolescents’ social and health issues

All the participants agreed that adolescents still lack basic necessities such as safe drinking water, electricity, cooking gas, and security. It was further expressed that lack of these basic necessities impedes academic achievements and employment opportunities. Hence, it causes unnecessary mental stress during this period of physical and mental development.

“Our children do not have basic facilities, and this is one of the barriers to their physical development and educational achievements.”–*(Parents & Community Leader)*

According to stakeholders, there has been a proliferation of private educational entities over the last two decades; however, these facilities do not provide quality education.

“Many newly constructed schools do not provide quality education.”–*(Madrassa & School Teacher)*

Parents and stakeholders informed that there are physical and sexual risks for adolescents, both boys and girls but more for girls.

“There are physical and sexual hazards for boys and girls, but more for girls.”- *(“Mother: Slum-III & Stakeholder)*

Parents restrict girls’ mobility and marry them immediately after puberty to protect them from physical and sexual abuse. Adolescents reported that mobility restrictions cause stress because these are imposed unexpectedly and without explanation. They also believed that before the age of 20, they are not prepared to handle in-laws and spousal relationships, including sexual encounters.

“Adolescents do not know anything about sex, sexual health, and marital relationships; they face difficulties in handling spousal relationships if married before age 20.”–*(Adolescents: Slum-1I, V & VIII & Healthcare Providers)*

Adolescent boys also believed that early marriage imposes a financial burden on married boys.

“Early marriage adds an economic burden on boys as they have to fulfill their families’ requirements.”–*(Male Adolescents: Slum-1V, VI & X)*

All respondents agreed that there are no social or sports facilities for adolescent recreation. Moreover, they are unaware of good and bad behaviors and practices. As a result, boys engage in a variety of bad behaviors and practices such as smoking, tobacco use, alcohol, and drugs, as well as criminal activities like drug selling, theft, mobile snatching, and physical and sexual harassment. Few even join criminal gangs.

“Boys get involved in criminal activities and even join criminal gangs.” *(Mother: Slum-V, NGO Representative, School Teacher)*

As a result of these risky behaviors, boys face friendship conflicts, injuries, road traffic accidents, and police arrests.

Adolescent girls and stakeholders reported a lack of understanding about sexual and reproductive health, as well as existing physical and sexual hazards. With increasing opportunities for interaction with boys through education and employment, many girls develop friendships with boys, which frequently leads to relationship conflict. As girl-boy friendship is still culturally unacceptable in this society, girls are unprepared to handle relationships with boys. Furthermore, adolescents keep these relationships secret as discussion about these relationships is considered a taboo subject.

“Girls develop relationships with boys secretly.”–*(Female Adolescents: Slum-1, III, VII & XI)*

Due to a lack of knowledge and skills to deal with such relationships and absence of guidance, many girls are subjected to bullying, blackmailing, and physical and sexual violence that sometimes ends in murder or suicide.

“Girls are the victims of violence by boyfriends, which in extreme cases results in murder or suicide.”–*(Female Adolescents: Slum-1, III, VII & XI, School & Madrassa Teachers and Healthcare providers)*

Despite being probed, parents did not acknowledge these girl-boy relationships, the resulting conflicts and unfortunate outcomes.

### 5. Adolescents’ decision-making process

Parents and adolescents revealed that, according to cultural norms, adolescents could only participate in decisions about their education, employment, or minor day-to-day issues like clothing and food. All major decisions, especially those related to marriage, are taken by parents, specifically the father. Parents were convinced that adolescents are incapable of making decisions.

“It will be disastrous if adolescents start making decisions.”—(*Mother: Slum-V, Political and Community Leaders)*

Most adolescents stated that they mostly discuss their problems with their friends and make decisions. Friends are especially preferred for discussing physical and sexual issues because adolescents believe that cultural norms prevent them from discussing these issues with their parents.

“Our cultural norms do not permit us to share our physical and sexual issues with parents.”–(*Boy: Slum-I, V, VI & VIII)*

Another reason for preferring friends is that adolescents believe that both parents work excessively hard to manage the large family size and therefore have no time for adolescents. There is no communication with fathers because most of them are the family’s sole breadwinners. As a result, they work longer hours and, in some cases, double shifts. This causes physical and mental exhaustion in fathers. As a result, they are usually in a bad mood and are unwilling to listen to the problems that adolescents face. Mothers are also busy with household chores, but they are reached out more often as they are welcoming.

### 6. Adolescents information needs, mechanism, and channels accessed

According to all the participants, social media networks such as Facebook, Instagram, and WhatsApp are the most frequently accessed information channels for both genders. In addition, they also get information from friends and television.

Parents, teachers and leaders were concerned that social media is disseminating inaccurate and socially unacceptable information, as well as undermining societal norms and value systems.

“Social media is spoiling the new generation.”–(Parents, School and madrassa teachers, Political and Community leaders)

All respondents agreed that adolescents are in desperate need of information, particularly on pubertal physical and sexual changes, minor puberty-related ailments, existing physical, social, and environmental hazards, healthy behaviors and practices, communication skills, career opportunities, the importance of good company, the harmful effects of addictions, and a correct version of an Islamic lifestyle, specifically concerning contraception. Adolescents should also be equipped with skills that will help them grow into responsible, healthy, and productive adults.

“Adolescents should be provided with information on pubertal physical and sexual changes, minor puberty-related ailments, existing physical, social, and environmental hazards, healthy behaviors and practices, communication skills, career opportunities, the importance of good company, the harmful effects of addictions, and a correct version of an Islamic lifestyle, specifically concerning contraception. They should also be taught skills for handling the physical, social and environmental hazards and enabling them to grow into responsible, healthy, and productive adults.”–*(All Male ^ Female Adolescents & Healthcare Providers)*

Adolescents believed that teachers should play a larger role in providing all this information because teachers and students have a friendlier relationship. However, for out-of-school adolescents, friends can play an active role.

## Discussion

**Fig 3:**
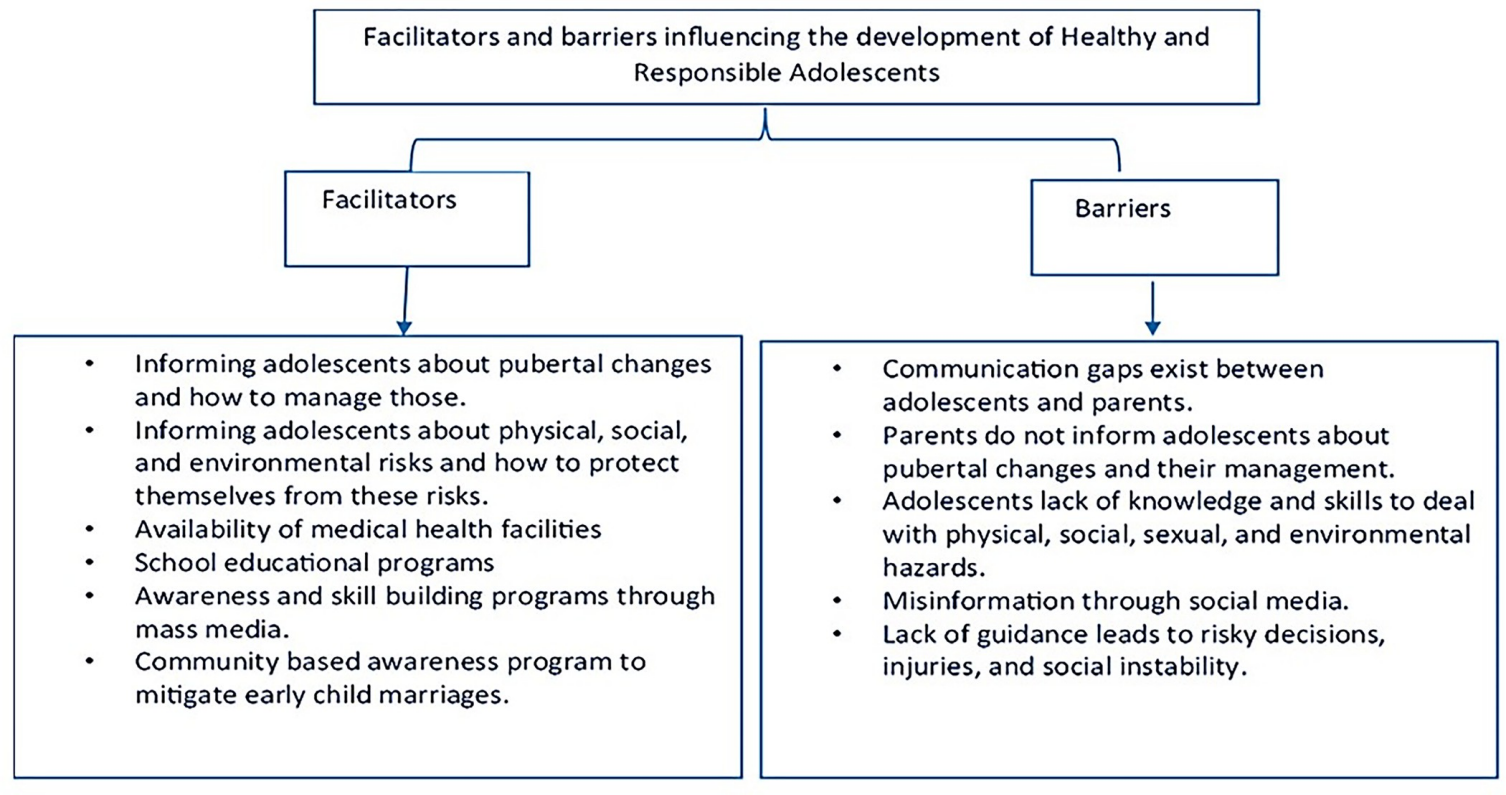
Summary of facilitators and barriers that influencing the development of healthy and responsible adolescents.

This study aimed to understand the lives of adolescents from the perspectives of stakeholders in terms of their characteristics, knowledge about pubertal changes, roles and responsibilities assigned to them, health and social issues they face, their decision-making processes, and mechanisms and channels they use to gather information. This study significantly contributes to the existing literature by providing a comprehensive exploration of the multifaceted experiences of adolescents in squatter settlements of Karachi, Pakistan. Unlike previous research which often focused narrowly on specific aspects of adolescent life, our study offers a holistic view, integrating perspectives from adolescents, their parents, and key stakeholders. We have uncovered intricate details about gender-specific roles, decision-making processes, and the unique challenges faced by adolescents in these communities. Particularly, our findings regarding the influence of gender norms on adolescents’ lives and the resultant disparities in education and social freedoms provide critical insights for policymakers and educators. Furthermore, the study sheds light on the pivotal role of communication gaps and the influence of social media, offering a nuanced understanding of the information channels utilized by adolescents. Such comprehensive insights are vital for developing targeted interventions and policies aimed at improving adolescent well-being in similar socio-cultural contexts (Refer to Fig 3).

Our study shows that all the participants were familiar with adolescent’s characteristics and their activities. The study discovered that adolescent boys and girls are assigned different roles and responsibilities; girls are made responsible for household chores, whereas boys are assigned outside tasks. This finding is consistent with evidence found from other studies [25, 26]. The study also identified that mobility restrictions imposed on girls have an impact on their ability to continue their education resulting in dropouts. Similar findings have been found in studies from countries with comparable social backgrounds [27, 28]. Gender norms, which are powerful pervasive beliefs about gender-based social roles and practices that are deeply embedded in social structures, are the source of these restrictions [27]. These gender norms are created by patriarchal power dynamics and maintained, in part, through self-surveillance as girls follow internalized gender scripts. Literature suggests strategies for overcoming gender stereotyping and reducing its effects on gender and social role formation, such as promoting a positive role for media and stabilizing a gender-balanced domestic environment [29]. It is necessary to raise awareness about the negative effects of gender roles. Studies have demonstrated that Behavior Change Communication (BCC) has changed gender norms [30]. Context-specific and tailored BCC interventions have the potential to overcome gender stereotyping and reduce its effects on gender roles, which can help to stabilize the gender-balanced domestic environment.

This study identified that a lack of necessities hinders the achievement of educational goals and, hence, career opportunities. Existing research also shows that poor socio-economic conditions have a negative impact on education and employment [31–35]. According to our study, adolescents are not adequately prepared to deal with existing physical, social, sexual, and environmental hazards. Moreover, communication gaps exist between adolescents and parents because discussing these risks is a taboo subject. Lack of knowledge and skills to deal with these challenges, as well as a lack of guidance, leads to risky decisions based on personal choices and peer advice. As a result of these poor decisions, adolescents face physical, social, sexual, and environmental issues, which results in injuries or even death. Studies show similar cases where adolescent girls are constantly subjected to bullying and sexual harassment [36–38] as a result of pubertal changes and aggression [36]. This emphasizes the importance of preparing adolescents to manage pubertal changes, take on new roles and responsibilities, and protect themselves from physical, social, and environmental risks. Studies have shown that parents, teachers, and healthcare providers are in the best position to communicate effectively with their children, listen to their needs, counsel, and provide guidance [39]. This clearly highlights the importance of raising awareness among parents, teachers, and healthcare providers about the risks that adolescents face, the significance of adolescent preparedness to protect themselves from these risks, and their role in this preparedness.

This study underscores the importance of acknowledging and empowering adolescents’ agency, advocating for interventions that not only address the external constraints imposed by gender norms and socio-economic conditions but also harness the inherent capabilities of adolescents to make informed decisions about their lives. Studies prove that adolescents can exhibit a dynamic role in navigating their health, education, and social interactions, contrary to the traditional view of them as passive participants in their developmental journey [40]. Our study showed that adolescents are obtaining information through easily accessible alternate pathways and mechanisms such as social media and television, as previously reported in other studies [40]. These information channels occasionally spread incorrect and socially unacceptable information that misleads adolescents. According to evidence, adolescents seek information to make informed decisions about their physical, sexual, and reproductive health, lifestyle, social relationships, communication, decision-making, and religion [41–49]. This insight calls for a paradigm shift in how we conceptualize and implement adolescent health and wellbeing programs, emphasizing a collaborative approach that values and leverages adolescents’ inputs and perspective.

The current study findings, as well as previous research, highlight the importance of empowering adolescents to effectively manage physical, sexual, social, and environmental challenges. Research propose several strategies to help adolescents manage their challenges including high-quality health worker training, adolescent-responsive facilities, and broad community engagement have all been proposed [2, 50]. We propose that the government should establish youth-led platforms to co-create blended learning content on core and transferable skills with parents, teachers, and healthcare providers. The participation of social, political, community, and religious leaders is critical in promoting and disseminating this content to adolescents in informal education while also advocating for its inclusion in formal education.

## Conclusion

The study concludes that the adolescents face several physical, sexual, reproductive, social, and environmental challenges during the adolescent period. Adolescents are ill-equipped to carry out their new roles and responsibilities, effectively manage the challenges they face, and protect themselves from physical and sexual abuse. Because of social norms, there is a communication gap between adolescents and their parents. The most popular information channels are social media platforms and friends.

Based on our findings, we recommend specific actions for key stakeholders to support adolescents’ development. Firstly, educators and policymakers should launch Behavior Change Communication (BCC) interventions aimed at dismantling gender stereotypes, thereby fostering a balanced domestic environment conducive to both boys’ and girls’ education and well-being. Secondly, it is imperative for parents, teachers, and healthcare providers to gain awareness about the multifaceted risks faced by adolescents, emphasizing their pivotal role in guiding and preparing the youth for these challenges. Lastly, we advocate for the creation of youth-led forums to collaboratively develop educational content alongside parents, educators, and health professionals, focusing on essential life skills. These efforts collectively can transform the demographic transition into a productive demographic dividend.

## Strengths and limitations of this study

This study stands out for its inclusive approach, incorporating a wide range of viewpoints from various stakeholders in the lives of adolescents across ten diverse areas of Karachi. This comprehensive perspective enriches the study’s findings, offering a multifaceted understanding of adolescent experiences. The finding of this study provides comprehensive information about adolescents where there is a dearth of research on adolescents specially those living and urban slums in LMICs including Pakistan. Within this scenario this study provides evidence to help policy makers and researchers to plan future interventions to improve adolescents’ health and social well-being. The study’s scope is confined to specific urban regions, impacting its generalizability. Future quantitative and qualitative studies can enhance our understanding of the breadth and depth of adolescent experiences in Pakistan and other LMICs.

## Declarations

### Ethical consideration & consent to participate

Ethical approval for this study was obtained from the Aga Khan University Ethical Review Committee (AKU-ERC). Written informed consent was provided by all study participants. Informed consent included permission to audio record the interviews and use anonymized quotes. Voluntary participation and the right to ask any questions and to decline participation at any time were emphasized during the data collection.

## References

[1] WHO. Global Accelerated Action for the Health of Adolescents (AA-HA!)—Guidance to Support Country Implementation. 2017.

[2] Patton GC, Sawyer SM, Santelli JS, Ross DA, Afifi R, Allen NB, et al. Our future: a Lancet commission on adolescent health and wellbeing. Lancet. 2016;387(10036):2423–78. Epub 20160509. doi: 10.1016/S0140-6736(16)00579-1 ; PubMed Central PMCID: PMC5832967. 27174304 PMC5832967

[3] Yoon Y, Eisenstadt M, Lereya ST, Deighton J. Gender difference in the change of adolescents’ mental health and subjective wellbeing trajectories. European Child & Adolescent Psychiatry. 2022:1–10. doi: 10.1007/s00787-022-01961-4 35246720 PMC8896070

[4] National Academies of Sciences E, Medicine, Health, Medicine D, Division of B, Social S, et al. In: Backes EP, Bonnie RJ, editors. The Promise of Adolescence: Realizing Opportunity for All Youth. Washington (DC): National Academies Press (US). Copyright 2019 by the National Academy of Sciences. All rights reserved.; 2019. 31449373

[5] Kuruvilla S, Bustreo F, Kuo T, Mishra CK, Taylor K, Fogstad H, et al. The Global strategy for women’s, children’s and adolescents’ health (2016–2030): a roadmap based on evidence and country experience. Bull World Health Organ. 2016;94(5):398–400. Epub 20160502. doi: 10.2471/BLT.16.170431 ; PubMed Central PMCID: PMC4850541. 27147772 PMC4850541

[6] Sheehan P, Sweeny K, Rasmussen B, Wils A, Friedman HS, Mahon J, et al. Building the foundations for sustainable development: a case for global investment in the capabilities of adolescents. Lancet. 2017;390(10104):1792–806. Epub 20170419. doi: 10.1016/s0140-6736(17)30872-3 . 28433259

[7] Organization WH. Adolescent health in the South-East Asia Region. Available from: https://www.who.int/southeastasia/health-topics/adolescent-health.

[8] Ali A, Khaliq A, Lokeesan L, Meherali S, Lassi ZS. Prevalence and predictors of teenage pregnancy in Pakistan: a trend analysis from Pakistan Demographic and Health Survey datasets from 1990 to 2018. International Health. 2021;14(2):176–82. doi: 10.1093/inthealth/ihab025 34013327 PMC8890806

[9] National Institute of Population Studies (NIPS) [Pakistan] and ICF. 2018. Pakistan Demographic and Health Survey 2017–18. Islamabad, Pakistan, and Rockville, Maryland, USA: NIPS and ICF.

[10] Sathar ZA, Lloyd CB, Diers J, Faizunnissa A. Adolescents and youth in Pakistan 2001–2002: a nationally representative survey. 2003.

[11] Sathar ZA, Kazi S. Women’s autonomy, livelihood and fertility: a study of rural Punjab. Women’s autonomy, livelihood and fertility: a study of rural Punjab. 1997.

[12] Hennink M, Rana I, Iqbal R. Knowledge of personal and sexual development amongst young people in Pakistan. Culture, health & sexuality. 2005;7(4):319–32. 16864206 10.1080/13691050500035367

[13] N R. Meri Awaz Suno: Analysis of Calls Received at Youth Help Line from January 2010-December 2011. Rozan NGO. 2012.

[14] Durrant V. Adolescent girls and boys in Pakistan: Opportunities and constraints in the transition to adulthood: Population Council Islamabad; 2000.

[15] Khan A. Adolescent and reproductive health in Pakistan: a literature review. 2001.

[16] ROZAN. Understanding Masculinities: A formative Research in Peri Urban Location of Rawalpindi Pakistan. 2010.

[17] Sawyer SM, Afifi RA, Bearinger LH, Blakemore SJ, Dick B, Ezeh AC, et al. Adolescence: a foundation for future health. Lancet. 2012;379(9826):1630–40. Epub 20120425. doi: 10.1016/S0140-6736(12)60072-5 . 22538178

[18] PAVHNA. Pakistan Voluntary Health and Nutrition Association: PAVHNA. 2000.

[19] Molyneux M. Adolescence: policy opportunities and challenges. UNICEF. 2020.

[20] Maguire M, Delahunt B. Doing a Thematic Analysis: A Practical, Step-by-Step Guide. 2017;9.

[21] Braun V, Clarke V. Thematic analysis. 2012. p. 57–71.

[22] Ziebland S, McPherson A. Making sense of qualitative data analysis: An introduction with illustrations from DIPEx (personal experiences of health and illness). Medical education. 2006;40:405–14. doi: 10.1111/j.1365-2929.2006.02467.x 16635119

[23] Forero R, Nahidi S, De Costa J, Mohsin M, Fitzgerald G, Gibson N, et al. Application of four-dimension criteria to assess rigour of qualitative research in emergency medicine. BMC Health Services Research. 2018;18(1):120. doi: 10.1186/s12913-018-2915-2 29454350 PMC5816375

[24] Lincoln YS, Guba EG. But is it rigorous? Trustworthiness and authenticity in naturalistic evaluation. New Directions for Program Evaluation. 1986;1986(30):73–84. doi: 10.1002/ev.1427

[25] Gracia P, Garcia-Roman J, Oinas T, Anttila T. Gender differences in child and adolescent daily activities: A cross-national time use study. Acta Sociologica. 2022;65(1):41–65. doi: 10.1177/00016993211008500

[26] Telford RM, Telford RD, Olive LS, Cochrane T, Davey R. Why Are Girls Less Physically Active than Boys? Findings from the LOOK Longitudinal Study. PLoS One. 2016;11(3):e0150041. Epub 2016/03/10. doi: 10.1371/journal.pone.0150041 ; PubMed Central PMCID: PMC4784873. 26960199 PMC4784873

[27] Bankar S, Collumbien M, Das M, Verma RK, Cislaghi B, Heise L. Contesting restrictive mobility norms among female mentors implementing a sport based programme for young girls in a Mumbai slum. BMC Public Health. 2018;18(1):471. doi: 10.1186/s12889-018-5347-3 29636027 PMC5894167

[28] Akyeampong K, Djangmah J, Oduro A, Seidu A, Hunt F. Access to Basic Education in Ghana: The Evidence and the Issues 2007.

[29] Hussain M, Naz A, Khan W, Daraz U, Khan Q. Gender Stereotyping in Family:An Institutionalized and Normative Mechanism in Pakhtun Society of Pakistan. SAGE Open. 2015;5(3):2158244015595258. doi: 10.1177/2158244015595258

[30] Pulerwitz J, Hui W, Arney J, Scott LM. Changing gender norms and reducing HIV and violence risk among workers and students in China. Journal of Health Communication. 2015;20(8):869–78. doi: 10.1080/10810730.2015.1018573 25950187

[31] Shlafer R, Hergenroeder AC, Jean Emans S, Rickert VI, Adger H, Spear B, et al. Adolescence as a critical stage in the MCH Life Course Model: commentary for the Leadership Education in Adolescent Health (LEAH) interdisciplinary training program projects. Maternal and child health journal. 2014;18(2):462–6. doi: 10.1007/s10995-013-1243-8 23435919 PMC5535310

[32] Johnson MK, Crosnoe R, Elder GH Jr. Insights on adolescence from a life course perspective. Journal of Research on Adolescence. 2011;21(1):273–80. doi: 10.1111/j.1532-7795.2010.00728.x 21483644 PMC3072576

[33] Group DBRCoCHDW. Starting early: a life-course perspective on child health disparities—research recommendations. Pediatrics. 2009;124(Suppl 3):S257. doi: 10.1542/peds.2009-1100O 19861478 PMC2976868

[34] Braveman P, Barclay C. Health disparities beginning in childhood: a life-course perspective. Pediatrics. 2009;124(Supplement_3):S163–S75. 19861467 10.1542/peds.2009-1100D

[35] Smith G, Hart C, Blane D. Hole D (1998),“Adverse Socioeconomic Conditions in Childhood and Cause Specific Adult Mortality: Prospective Observational Study”. British Medical Journal. 316:1631–35. doi: 10.1136/bmj.316.7145.1631 9603744 PMC28561

[36] Vega-Gea E, Ortega-Ruiz R, Sánchez V. Peer sexual harassment in adolescence: Dimensions of the sexual harassment survey in boys and girls. International Journal of Clinical and Health Psychology. 2016;16(1):47–57. doi: 10.1016/j.ijchp.2015.08.002 30487850 PMC6225015

[37] Pepler DJ, Craig WM, Connolly JA, Yuile A, McMaster L, Jiang D. A developmental perspective on bullying. Aggressive Behavior: Official Journal of the International Society for Research on Aggression. 2006;32(4):376–84.

[38] Shute R, Owens L, Slee P. Everyday Victimization of Adolescent Girls by Boys: Sexual Harassment, Bullying or Aggression? Sex Roles. 2007;58:477–89. doi: 10.1007/s11199-007-9363-5

[39] Luxford K, Safran DG, Delbanco T. Promoting patient-centered care: a qualitative study of facilitators and barriers in healthcare organizations with a reputation for improving the patient experience. International Journal for Quality in Health Care. 2011;23(5):510–5. doi: 10.1093/intqhc/mzr024 21586433

[40] Tremolada M, Silingardi L, Taverna L. Social Networking in Adolescents: Time, Type and Motives of Using, Social Desirability, and Communication Choices. International journal of environmental research and public health. 2022;19(4). Epub 2022/02/26. doi: 10.3390/ijerph19042418 ; PubMed Central PMCID: PMC8872287. 35206605 PMC8872287

[41] Skinner H, Biscope S, Poland B, Goldberg E. How adolescents use technology for health information: implications for health professionals from focus group studies. Journal of medical Internet research. 2003;5(4):e32. Epub 2004/01/10. doi: 10.2196/jmir.5.4.e32 ; PubMed Central PMCID: PMC1550577. 14713660 PMC1550577

[42] Pfeifer JH, Masten CL, Moore WE III, Oswald TM, Mazziotta JC, Iacoboni M, et al. Entering adolescence: resistance to peer influence, risky behavior, and neural changes in emotion reactivity. Neuron. 2011;69(5):1029–36. doi: 10.1016/j.neuron.2011.02.019 21382560 PMC3840168

[43] Chein J, Albert D, O’Brien L, Uckert K, Steinberg L. Peers increase adolescent risk taking by enhancing activity in the brain’s reward circuitry. Wiley Online Library; 2011. 10.1111/j.1467-7687.2010.01035.x PMC3075496 21499511

[44] Gardner M, Steinberg L. Peer influence on risk taking, risk preference, and risky decision making in adolescence and adulthood: an experimental study. Developmental psychology. 2005;41(4):625. doi: 10.1037/0012-1649.41.4.625 16060809

[45] Smith AR, Chein J, Steinberg L. Peers increase adolescent risk taking even when the probabilities of negative outcomes are known. Developmental psychology. 2014;50(5):1564. doi: 10.1037/a0035696 24447118 PMC4305434

[46] Mann L, Harmoni R, Power C. Adolescent decision-making: the development of competence. Journal of Adolescence. 1989;12(3):265–78. doi: 10.1016/0140-1971(89)90077-8 2687339

[47] Marques A, Loureiro N, Avelar-Rosa B, Naia A, Matos MGd. Adolescents’ healthy lifestyle. Jornal de Pediatria. 2020;96(2):217–24. doi: 10.1016/j.jped.2018.09.002 30393010 PMC9432147

[48] Organization WH. Action plan for the prevention and control of noncommunicable diseases in the WHO European Region. World Health Organization. Regional Office for Europe, 2016.

[49] Kelly SA, Melnyk BM, Jacobson DL, O’Haver JA. Correlates among healthy lifestyle cognitive beliefs, healthy lifestyle choices, social support, and healthy behaviors in adolescents: implications for behavioral change strategies and future research. Journal of pediatric health care. 2011;25(4):216–23. doi: 10.1016/j.pedhc.2010.03.002 21700136

[50] Adolescent friendly health services for adolescents living with HIV: from theory to practice. Technical Brief. Geneva, Switzerland: 2019.

