# Supplementary material for: Are we preparing healthy & responsible adolescents? Exploratory qualitative study to understand the health and social issues of adolescent living in Karachi, Pakistan
Source: PLOS Glob Public Health. 2024 Oct 15;4(10):e0003736. doi: 10.1371/journal.pgph.0003736 (PMC11478873; doi:10.1371/journal.pgph.0003736)
Supplement: Attachment — Submitted filename: My Remarks.docx [file pgph.0003736.s002.docx]

**REVIEWER'S REMARKS ON THE MANUSCRIPT TITLED** *“ARE WE PREPARING HEALTHY & RESPONSIBLE ADOLESCENTS? EXPLORATORY QUALITATIVE STUDY FROM KARACHI, PAKISTAN” PGPH-D-23-02041R1*

| **Sl. No** | **Authors data** | **Line no** | **My (reviewer) Comments** |
| --- | --- | --- | --- |
| 1. | Are We Preparing Healthy & Responsible Adolescents? Exploratory Qualitative Study  from Karachi, Pakistan | 1 | The phrase in the title ought to be broad and indicate your objectives. Reframe in light of your themes, research design, and study area. |
| 2. | **Keywords:** Adolescents Roles, Preparedness, Information, Communication Channel | 60 | MeSH based keywords need to add |
| 3. | In Karachi, Pakistan, squatter settlements lack basic amenities such as health, education, sports, and other social services and infrastructure which are essential for adolescents’ normal growth and upbringing. This study aimed to understand the lives of adolescents in Karachi, Pakistan, by gaining the perspectives from teenagers, parents, and stakeholders regarding adolescents’ characteristics, activities they are involved in, health and social issues they face, their decision-making process, and mechanism and channels they use to gather information. | 109-113 | The last paragraph of the introduction should include a mention of the problem statement or study scope. The goals of this study should be added rather than being the aim. |
| 4. | The study used a qualitative descriptive approach to understand the lives of adolescents through adolescents’ and stakeholders’ perspectives in terms of adolescents’ characteristics, their knowledge about pubertal changes, roles and responsibilities assigned to them, health, and social issues they face, their decision-making processes, and the techniques and channels they use to gather information | 116-119 | Create a separate statement for the study's purpose and design. |
| 5. | The primary objective of ‘Sukh’ was to increase contraceptive use by 15% among Karachi, Sindh, Pakistan’s 100 million underserved peri-urban population. Additionally, the project also explored the lives of adolescents. The study was conducted in ten squatter settlements in four towns of Karachi where the ‘Sukh’ field-based health and social centers existed – Table 1. Participants included, a diverse set of key stakeholders - adolescents aged 16 to 18, adolescents parents, government sports and youth affairs officers, political leaders, social activists, NGO administrators, educational leaders from public and private sectors, healthcare professionals, and community health workers. A purposive sampling approach, detailed in Table 1, was designed to capture a wide range of perspectives based on gender, geographic location, and social roles, thus providing a comprehensive understanding of the multifaceted adolescent experience in these communities. This diverse participant pool offered varied viewpoints, enriching our study with insights from different socioeconomic, cultural, and professional backgrounds | 124-134 | Rather than using take, describe the steps for choosing samples here. I suggest doing away with the tables. |
| 6. | Interview guide was pilot-tested with five participants. | 139 | For the following data collection process, any alterations or reflections need to be noted in writing. |
| 7. | Data were collected iteratively, between 01st December 2014 to 31st January 2017 | 145 | The data was gathered earlier than seven years. Do you believe the information is relevant to the current situation? because many modifications have occurred since COVID. How are you going to defend it? |
| 8. | Data Collection | 135 | There needs to be a focus group and interview checklist. Here's an illustration of a COREQ checklist. When collecting data, were there guidelines that you followed? |
| 9. | Applicable data protection regulations | 154 | Please describe it |
| 10. | Data Analysis | 156 | The amount of data is enormous. Have you used any programme for theme analysis? utilised software such as Atlas TI, to name a few. Please mention that… |
| 11. | Results | 171 | My suggested order for the results is as follows: 1. Respondents' sociodemographic information  2. The frequency of themes and subthemes (that is, the number of responses to each specific theme)  3. Themes with an objective and their subthemes  4. The verbatims of the FGDs and IDIs Key Informant participants must be included under each subtheme. There must be at least two sentence interpretations and at least four to five verbatims under each subtheme.  5. Repossess number need to refer. For instance, (Male adolescent, Age, Number of Respondents) |
| 12. | 55.3% (n=105) of adolescents who participated in FGDs were females | 173-174 | Parentage does not begin a sentence. I would advise rephrasing the sentence. |
| 13. | Adolescents Characteristics | 182 | Not within the theme It ought to be explained in a table under the section titled "Respondents' sociodemographic details." |
| 14. | *(Female Adolescents, Slum I, V & VIII & Lady Health Worker).* | 195 | The opinions of the respondents differed on this. Need to change it. |
| 15. | Discussion | 310 | Please reframe according to the results. |
| 16. | Summary of key facilitators and barriers required for the development of healthy and responsible adolescents are given in Figure-3. | 381-382 | Lacking directions for the future in the figure, and it ought to be addressed in light of current research. |
| 17. | Strengths and Limitations of this study | 400 | Rephrasing limitations is necessary. |

Thanking You, All the Best.
