# Supplementary material for: Are we preparing healthy & responsible adolescents? Exploratory qualitative study to understand the health and social issues of adolescent living in Karachi, Pakistan
Source: PLOS Glob Public Health. 2024 Oct 15;4(10):e0003736. doi: 10.1371/journal.pgph.0003736 (PMC11478873; doi:10.1371/journal.pgph.0003736)
Supplement: Attachment — Submitted filename: Response to Reviewers.docx [file pgph.0003736.s003.docx]

Response to Reviewers *“ARE WE PREPARING HEALTHY & RESPONSIBLE ADOLESCENTS? EXPLORATORY QUALITATIVE STUDY FROM KARACHI, PAKISTAN” PGPH-D-23-02041R1*

| **S. No** | **Journal requirement** | **Authors comments** |
| --- | --- | --- |
| 1. | Please amend your detailed Financial Disclosure statement. This is published with the article. It must therefore be completed in full sentences and contain the exact wording you wish to be published. | 426 -427 |
|  | **Editor comments** |  |
| 2. | We have noticed that you have uploaded Supporting Information files, but you have not included a list of legends. Please add a full list of legends for your Supporting Information files after the references list | Attached |
| **S. No** | **Reviewer Comments** | **Authors comments** |
| 1. | The phrase in the title ought to be broad and indicate your objectives. Reframe in light of your themes, research design, and study area. | Address line no: 1-2 |
| 2. | MeSH based keywords need to add | Text added line no : 60 -61  “health and social issues, Decision-making , Adolescent health” |
| 3. | The last paragraph of the introduction should include a mention of the problem statement or study scope. The goals of this study should be added rather than being the aim. | Text edited Line no :112-113 |
| 4. | Create a separate statement for the study's purpose and design. | Text edited line no : 116-117 |
| 5. | Rather than using take, describe the steps for choosing samples here. I suggest doing away with the tables. | Feedback incorporated. Step added  (bullet form) line no 123-136 |
| 6. | For the following data collection process, any alterations or reflections need to be noted in writing. | Text added line no. 145-146 |
| 7. | The data was gathered earlier than seven years. Do you believe the information is relevant to the current situation? Because many modifications have occurred since COVID. How are you going to defend it? | Comments: Appreciating the reviewer’s feedback, it is important to acknowledge that the current situation of adolescents living in urban slums of Low-and-Middle-Income-Countries (LMICs) including Pakistan has not changed. The parenting norms have remained the same and no major modifications have occurred in the health and social systems during the last several decades. Moreover, there is mushrooming of urban slums in LMICs. Still there is a dearth of research on adolescents specially those living and urban slums. Furthermore, because high fertility countries like Pakistan have been facing ‘Youth Bulge’. Consequently, these countries currently have the highest adolescent population. Within in this scenario this data is extremely valuable and still relevant and provide evidence to help policy makers and researchers to plan future interventions to improve adolescents’ health social well-being. |
| 8. | There needs to be a focus group and interview checklist. Here's an illustration of a COREQ checklist. When collecting data, were there guidelines that you followed? | Addressed. COREQ checklist were followed and attached in Annexure. |
| 9. | Please describe it |  |
| 10. | The amount of data is enormous. Have you used any programme for theme analysis? utilised software such as Atlas TI, to name a few. Please mention that… | Addressed Line no 165  QSR-Nvivo 12 software. |
| 11. | My suggested order for the results is as follows: 1. Respondents' sociodemographic information  2. The frequency of themes and subthemes (that is, the number of responses to each specific theme)  3. Themes with an objective and their subthemes  4. The verbatims of the FGDs and IDIs Key Informant participants must be included under each subtheme. There must be at least two sentence interpretations and at least four to five verbatims under each subtheme.  5. Repossess number need to refer. For instance, (Male adolescent, Age, Number of Respondents) | Comments: We encourage reviewer comments and edits made where necessary 181-187 and 187 -188 |
| 12. | Parentage does not begin a sentence. I would advise rephrasing the sentence. | Text modified according to feedback. 176-179 |
| 13. | Not within the theme It ought to be explained in a table under the section titled "Respondents' sociodemographic details." | Moved 182- 188 |
| 14. | The opinions of the respondents differed on this. Need to change it. | Edits made 192-193 |
| 15. | Please reframe according to the results. | Text edited 311-314 |
| 16. | Lacking directions for the future in the figure, and it ought to be addressed in light of current research. | Text edited 381-382 |
| 17. | Rephrasing limitations is necessary. | 400-402 |
