# Supplementary material for: Are we preparing healthy & responsible adolescents? Exploratory qualitative study to understand the health and social issues of adolescent living in Karachi, Pakistan
Source: PLOS Glob Public Health. 2024 Oct 15;4(10):e0003736. doi: 10.1371/journal.pgph.0003736 (PMC11478873; doi:10.1371/journal.pgph.0003736)
Supplement: Attachment — Submitted filename: Response-Reviewer 13.8.2024.docx [file pgph.0003736.s004.docx]

| Editor comments | Response |
| --- | --- |
| Please amend your detailed online Financial Disclosure statement. This is published with the article. It must therefore be completed in full sentences and contain the exact wording you wish to be published. | Text added |
| . Please upload the latest version of your revised manuscript as the main article file, with the item type 'Manuscript,' ensuring that it does not contain any tracked changes or highlighting. This will be used in the production process if your manuscript is accepted. | Manuscript uploaded with proofread |
| Please provide separate figure files in .tif or .eps format only and ensure that all files are under our size limit of 10MB. | Files added |
| Text added | Please include a separate legend or caption for Figure 3 in your manuscript. |
