# Supplementary material for: Are we preparing healthy & responsible adolescents? Exploratory qualitative study to understand the health and social issues of adolescent living in Karachi, Pakistan
Source: PLOS Glob Public Health. 2024 Oct 15;4(10):e0003736. doi: 10.1371/journal.pgph.0003736 (PMC11478873; doi:10.1371/journal.pgph.0003736)
Supplement: Attachment — Submitted filename: Response-Reviewer Comments-PG_07022024.docx [file pgph.0003736.s001.docx]

**Response to Reviewers**

**Article Title:**

**Reference: PGPH-D-23-02041**

| **Reviewer Number** | **Comment** | **Response** | **Line Number** | **Page Number** |
| --- | --- | --- | --- | --- |
| Reviewer 1 | The paper could benefit from a stronger discussion section beyond confirming what other studies have foun0064. What is the specific value add of this data? What are the key urgent messages? In addition, the paper could benefit from a theoretical basis... Grounding the discussion in theory will further strengthen the paper. | The discussion has been revised however, for the second part of the comment, please note that this was a exploratory qualitative descriptive study therefore no grounding theory was applicable. | 309-381 | 8-11 |
| Reviewer 1 | The concept of adolescents' agency seems to be emerging but not very well discussed in the paper. Expounding on this could add much value to the paper, acknowledging the role of adolescents in their own health and wellbeing Vs positioning them as passive beings in their wellbeing and development. | We have edited and strengthened the discussion. Thank you for your valuable comments and insights. | 309-381 | 8-11 |
| Reviewer 1 | The authors could add some more helpful detail to methods section for instance, how many adolescents were interviewed? How many were male, female?... What languages were used in the discussions? Were there any particular play based or other specific approaches used in the discussions? | Methods section is revised. The required information has been added to the results section as Figure 2 and 3.  There was no play based or other specific approaches used. | 114-170 | 4-5 |
| Reviewer 1 | There is need for some specificity on who the recommendations are for. Who should do what? They are very general. Highlighting potential areas for future research might also be useful. | This section has been thoroughly edited with specific recommendations as well as future research areas. | 378-382 | 10-11 |
| Reviewer 2 | Please specify the qualitative method/methodology that guided the study design. For example, qualitative description. | We added the specific qualitative method used. | 114-170 | 4-5 |
| Reviewer 2 | Use a language that suggests that authors are reporting the perspectives of the three groups rather than taking for granted what participants stated. | Point has been noted and edits have been made in the results section throughout. | 171-308 | 5-8 |
| Reviewer 2 | Abstract conclusion does not provide the key practical and research implications that can be derived from the study conducted. | Edited/revised to ensure it highlights the key practical and research implications | 53-59 | 2 |
| Reviewer 2 | A reference is needed for the claim made in the third sentence of the first paragraph. Overall, this section is well-structured. | It is the same reference as 3 | 74 | 3 |
| Reviewer 2 | Like quantitative research, qualitative research is a research inquiry. Thus, the specific qualitative research method that guided the study design, if any, should be indicated and justified. | It is exploratory descriptive qualitative study design | 116 | 4 |
| Reviewer 2 | Please specify the purposeful sampling strategy employed... | Edited. | 130 | 4 |
| Reviewer 2 | Why did the authors claim that data collection continued until achieving theoretical saturation if this study does not aim to develop a substantive theory based on qualitative data? | Appropriate changes are made. | 153 | 4 |
| Reviewer 2 | A heading should be used for data collection and for data analysis. | A data collection heading is already provided. Data analysis subheading is added. | 156 | 4 |
| Reviewer 2 | Rigor has to be demonstrated rather than suggested. Authors are supposed to state how the criteria of rigor were implemented. | Well noted. The rigor section has been edited with proper details. | 164-170 | 5 |
| Reviewer 2 | Table 2 does not appear to provide characteristics of participants, but the total number of participants in each group. | Correction has been made. | 176 | 5 |
| Reviewer 2 | Data analysis does not appear to have yielded themes but categories or topics within which the relevant data were sorted. | We have edited the themes to the original themes that were yielded from the data analysis. | 182,189, 204 | 5-7 |
| Reviewer 2 | In qualitative research, researchers are encouraged to report the diversity of views within and across groups involved in the study. | We have revised methods to add following details of the participants:  Adolescents aged 16 to 18, their parents, and a variety of key stakeholders - government sports and youth affairs officers, political leaders, social activists, NGO administrators, educational leaders from public and private sectors, healthcare professionals, and community health workers. This purposive sampling approach, detailed in Table 1, was designed to capture a wide range of perspectives based on gender, geographic location, and social roles, thus providing a comprehensive understanding of the multifaceted adolescent experience in these communities. This diverse participant pool offered varied viewpoints, enriching our study with insights from different socioeconomic, cultural, and professional backgrounds. | 114-170 | 4-5 |
| Reviewer 2 | Please highlight in this section (preferably in the first paragraph) the study contribution, what it added to the current literature. | Added a paragraph about what the study contributes to the current literature | 311-324 | 8-9 |
| Reviewer 2 | Please add study limitations and future directions. Make the conclusions more concise. | We have added a section on limitations and strengths as well as future directions. Conclusion has been edited. | 384-406 | 11 |
| Reviewer | Quotes are needed to support the last interpretation (final paragraph) in this (results) section. | Added | 302-307 | 8 |
